# Raman Spectroscopy and Machine Learning Enables Estimation of Articular Cartilage Structural, Compositional, and Functional Properties

**DOI:** 10.1007/s10439-023-03271-5

**Published:** 2023-06-16

**Authors:** Eslam Shehata, Ervin Nippolainen, Rubina Shaikh, Ari-Petteri Ronkainen, Juha Töyräs, Jaakko K. Sarin, Isaac O. Afara

**Affiliations:** 1 Department of Technical Physics, University of Eastern Finland, Kuopio, Finland; 2 Diagnostic Imaging Center, Kuopio University Hospital, Kuopio, Finland; 3 Science Service Center, Kuopio University Hospital, Kuopio, Finland; 4 School of Information Technology and Electrical Engineering, The University of Queensland, Brisbane, Australia; 5 Department of Medical Physics, Medical Imaging Center, Pirkanmaa Hospital District, Tampere, Finland

**Keywords:** Osteoarthritis, Biomechanics, Raman spectroscopy, Machine learning, Classification, Regression

## Abstract

**Objective:**

To differentiate healthy from artificially degraded articular cartilage and estimate its structural, compositional, and functional properties using Raman spectroscopy (RS).

**Design:**

Visually normal bovine patellae (*n* = 12) were used in this study. Osteochondral plugs (*n* = 60) were prepared and artificially degraded either enzymatically (via Collagenase D or Trypsin) or mechanically (via impact loading or surface abrasion) to induce mild to severe cartilage damage; additionally, control plugs were prepared (*n* = 12). Raman spectra were acquired from the samples before and after artificial degradation. Afterwards, reference biomechanical properties, proteoglycan (PG) content, collagen orientation, and zonal (%) thickness of the samples were measured. Machine learning models (classifiers and regressors) were then developed to discriminate healthy from degraded cartilage based on their Raman spectra and to predict the aforementioned reference properties.

**Results:**

The classifiers accurately categorized healthy and degraded samples (accuracy = 86%), and successfully discerned moderate from severely degraded samples (accuracy = 90%). On the other hand, the regression models estimated cartilage biomechanical properties with reasonable error (≤ 24%), with the lowest error observed in the prediction of instantaneous modulus (12%). With zonal properties, the lowest prediction errors were observed in the deep zone, i.e., PG content (14%), collagen orientation (29%), and zonal thickness (9%).

**Conclusion:**

RS is capable of discriminating between healthy and damaged cartilage, and can estimate tissue properties with reasonable errors. These findings demonstrate the clinical potential of RS.

**Supplementary Information:**

The online version contains supplementary material available at 10.1007/s10439-023-03271-5.

## Introduction

Articular cartilage (AC) possesses a limited healing capacity and thus, early detection and treatment of injuries are of paramount importance. Current clinical approaches for AC injury diagnosis are based on radiography and magnetic resonance imaging, which possess relatively low sensitivity to early degenerative changes and correlate only moderately with clinical endpoints [1]. In case of injury, the patient may proceed to arthroscopic repair surgery, allowing also the inspection of the overall health of the joint [2]. However, conventional arthroscopy relies on the surgeon’s visual and tactile assessment of the tissue, which results in subjective and poorly reproducible outcomes [2]. Recently, several techniques including ultrasonography [3], high frequency [4] and intra-articular [5] ultrasound, optical coherence tomography [6, 7], near-infrared (NIR) [8–10], mid-infrared [11, 12], and RS [13–16] have been proposed as potential candidates for addressing the limitations of conventional arthroscopic approaches.

RS is based on the energy shift between the incident and inelastically scattered photons off the electric dipole of a molecule. Applications of RS in biomedical engineering are rapidly increasing [17]. Various studies [17, 18] have mapped Raman peaks in biological tissues to their underlying structures (e.g., functional groups, bonding types, and molecular conformations). Raman peaks are relatively narrow, easy to resolve, and sensitive to molecular structure, conformation, and environment, thus resulting in high chemical specificity [19]. In addition to being a relatively simple, reproducible, and non-destructive technique, RS requires a small sample size with minimal sample preparation. RS is not affected by water interference, which is abundant in biological tissues [20]. Several studies have explored the potential of RS for characterizing the integrity of AC [13, 21]. However, only a few studies have focused on estimating cartilage biomechanical, compositional, or structural [22] properties via RS [23, 24].

In 2011 [14], Esmonde-White et al. conducted a proof-of-concept study to assess the potential of RS for the arthroscopic assessment of joint tissues by adapting a custom-designed Raman fibre optic probe to examine the knees of human cadavers and tissue phantoms. In 2019 [16], RS was used to probe the biochemical composition of the synovial fluid of 40 patients who had different stages of  osteoarthritis (OA). The study demonstrated the capacity of RS for the severity assessment of joint disease. While the results were promising, the approach provides no localized information about the disease. A prior study [25] indicates that Raman spectra can reliably detect disease-related changes in equine cartilage samples via correlation with the International Cartilage Repair Society (ICRS [26]) scores. However, limited to no research has been conducted to investigate the relationship between Raman spectra and AC biomechanical properties.

Although RS is information-rich, spectral analysis is either time-consuming and complicated or limited in bandwidth. Recent advances in computational hardware have allowed the use of machine learning (ML) techniques, which have superior performance related to conventional chemometric methods [27], allowing further utilization of RS. In this study, we hypothesize that RS, coupled with ML, can differentiate healthy from degraded AC, and estimate its structural, compositional, and functional integrity. To test this hypothesis, we collected Raman spectra from healthy and degraded AC with varying degrees of degeneration, along with reference properties for the degraded samples. Then, we developed ML models to classify healthy and degraded samples and estimate the correlation between the Raman spectra and the aforementioned cartilage properties.

## Materials and Methods

**Fig. 1:**
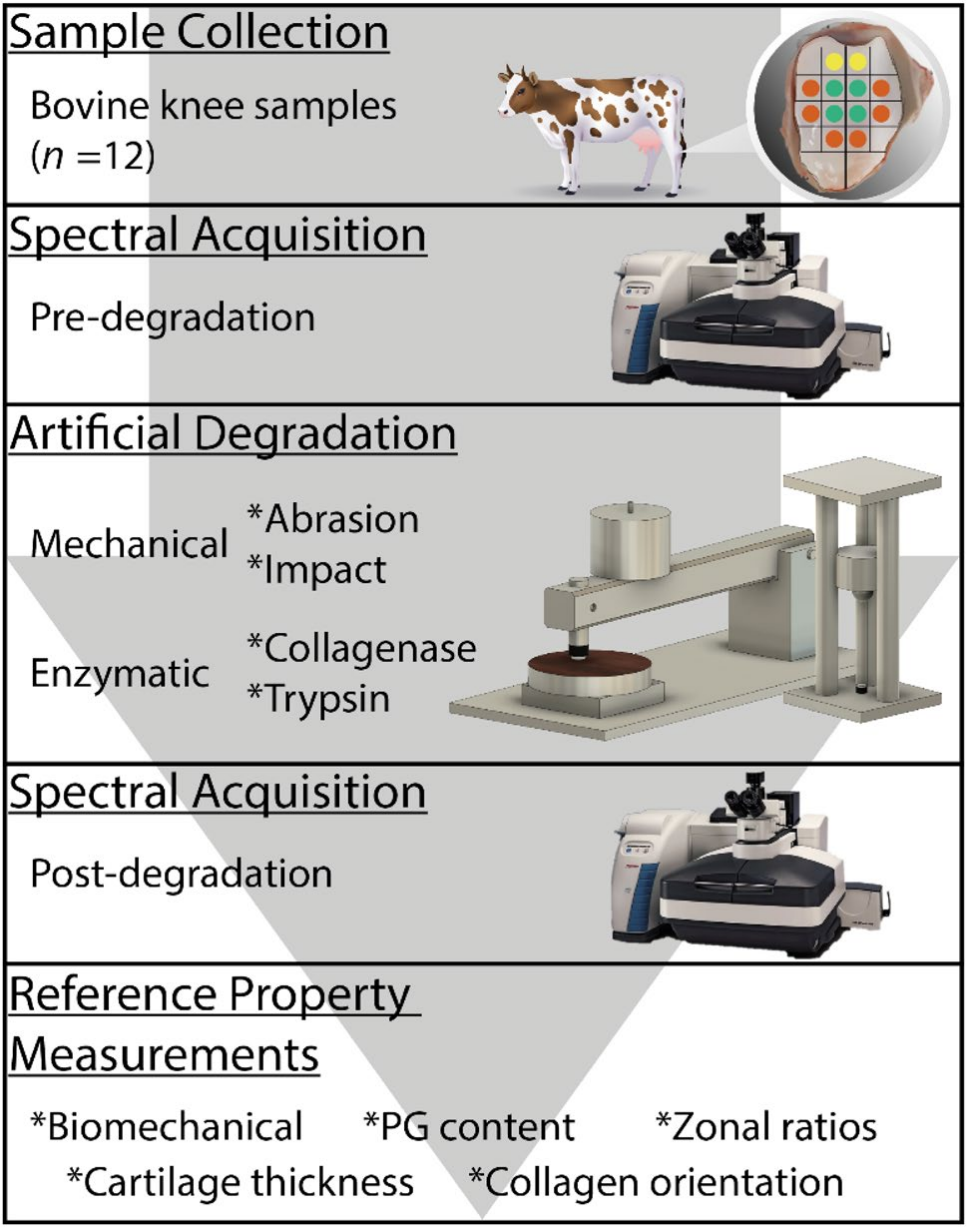
Experimental design and data collection workflow (*PG* proteoglycan). Panel 1 (yellow = CNTRL, green (top = IL, bottom = SA), orange (top = COL24h, middle = COL90m, bottom = TRP30m)

Fresh bovine (age 14–22 months) knee joints (*n = *10) obtained from a local abattoir were used in this study; thus, no ethical permission was required. The patella of each knee was divided vertically into two halves (i.e., medial and lateral), only visually healthy (i.e., without discolouration, surface roughness, or ruptures) halves (*n* = 12), were used. From each half, a total of six samples were prepared (Fig. 1): one for control, two for mechanical degradation, and three for enzymatic degradation.

In preparation for artificial degradation, cylindrical osteochondral samples for the mechanical damage group (*n* = 24) were extracted using a biopsy punch (*d* = 7 mm), while larger rectangular samples (10 × 15 mm^2^) were extracted for the enzymatic damage group (*n* = 36). This is to minimize lateral penetration of the enzyme during incubation (no lateral penetration was observed in histological imaging). Following enzymatic treatment, cylindrical osteochondral samples (*d* = 7 mm) were then obtained from the centre of the rectangular samples. Control cylindrical osteochondral samples (*n* = 12) were also extracted for reference measurements.

Raman spectra were acquired from each sample three times using a Thermo Fisher Scientific’s (Madison, WI, USA) DXR2xi Raman confocal microscope. The spectra were collected from the centre of each sample using a 10 × objective with a 50 µm confocal pinhole. A 30-mW powered laser with a 785 nm central wavelength was used to minimize fluorescence. The device was configured to obtain wavelength shifts in the range of 50 cm^−1^ to 3400 cm^−1^. The exposure time was 0.5 s with 120 accumulations [13]. These measurements were performed before and after the initiation of artificial degradation, where the measurements conducted before degradation were used as control references, in addition to the control samples. All samples were stored in PBS during the measurements and were washed thoroughly with 4 °C PBS within 1 hour before testing to protect them from natural enzymatic degradation.

### Mechanical Damage

Two types of mechanical injury models were applied in this study to simulate traumatic joint injuries. For the first injury model, a sub-group of cylindrical osteochondral samples (*n = *12) were subjected to impact loading (IL), while for the second injury model, another sub-group (*n* = 12) was subjected to surface abrasion (SA).

The IL injury was induced using a custom-made drop tower (Fig. 1) to create minor chondral cracks on the cartilage surface as described by Kokkonen et al. [28] Briefly, a steel ball (*d* = 10 mm) attached to a stainless-steel impactor (*m* = 200 g) was allowed to free fall from a height of 75 mm onto the sample to impart an energy of 0.147 J. The impactor was lifted from the sample immediately after the impact to prevent creep deformation.

The SA damage was performed with a custom-made grinding tool (Fig. 1). The surface of each sample was abraded under constant stress (4 kPa) by rotating (180°, CW and CCW) a metal plate with P80 sandpaper (corresponding to a particle size of 200 µm, Mirox P80, Mirka Oy, Uusikaarlepyy, Finland).

All samples were rinsed in phosphate-buffered saline (PBS) for 1 hour, immediately after each respective mechanical injury to allow cartilage recovery.

### Enzymatic Degradation

Samples for enzymatic degradation were further divided into three sub-groups to simulate early and advanced OA. The first two sub-groups were subjected to Collagenase D (0.1 mg/ml, Sigma-Aldrich, Inc., St. Louis, MO, USA) treatment for 90 min (COL90m, *n* = 12) and 24 h (COL24h, *n* = 12), respectively. While the third sub-group was subjected to trypsin (0.5 mg/ml, T4299, Sigma-Aldrich, Inc., St. Louis, MO, USA) digestion for 30 min (TRP30m, *n* = 12). Collagenase D was used to degrade the collagen network [29], while trypsin was employed to deplete PGs [30], with minor collateral effect on the collagen network. COL24h group represented advanced OA, while COL90m and TRP30m represented the earlier stages of the disease. All samples were incubated at 37 °C and 5% CO_2_ in PBS containing the enzymes and supplemented with antibiotics, including Penicillin (100 U/ml)–Streptomycin (100 µg/ml)–Amphotericin B (0.25 µg/ml, Sigma-Aldrich, Inc., St. Louis, MO, USA). Similar to the mechanical damage group, all samples were rinsed in PBS solution.

### Reference Measurements

AC structure can be divided into three zones (superficial, mid, and deep zones) according to its depth-wise collagen fibre orientation [31]. Type II collagen fibrils in AC matrix are organized in an inverted U shape network (also called the Benninghoff arcades [32]), where they are parallel to the surface in the superficial zone and normal to it in the deep zone. This specific orientation enables the network to withstand tensile loading and maintain the cartilage volume and shape. On the other hand, PGs, which exhibit increasing content with tissue depth, are responsible for the tissue’s equilibrium modulus as well as its capability of returning to its original shape after deformation. To characterize the samples in this study, reference measurements were conducted to obtain the aforementioned fundamental tissue properties.

Biomechanical measurements were conducted using a custom-made high-precision (resolution 0.1 µm, 0.005 N) material testing device [28], equipped with a cylindrical indenter (*d* = 0.7 mm). Measurements were performed at the centre of the samples while submerged in PBS. Prior to measurement, the bone end of each osteochondral sample was glued to the bottom of the measurement chamber. A goniometer (#55-841, Edmund Optics, Inc., Barrington, NJ, USA) was used to adjust the perpendicularity between the cartilage surface and the indenter tip. First, the samples were preconditioned using a cyclic 2% strain (4 full cycles). A stress-relaxation protocol was then implemented to determine both the equilibrium modulus (*E*_eq_) and the instantaneous modulus (*E*_inst_). The stress-relaxation protocol consisted of three steps. In each step, the cartilage was compressed 5% of its remaining thickness, with a compression rate of 100% cartilage thickness/s. The sample was left to relax for 900 s between each step. The aforementioned moduli were calculated from the average of the second and the third step. At the final compression step, the dynamic modulus (*E*_dyn_) was measured by a sinusoidal dynamic test which was conducted at frequencies of 0.1, 0.5, 1.0, and 2.0 Hz. The sinusoidal test was done for five cycles with a strain amplitude equal to 2% of the remaining cartilage thickness. An elastic–isotropic model was adopted to calculate all the moduli mentioned above [33] with a Poisson’s ratio of 0.2 for equilibrium modulus and 0.5 for instantaneous and dynamic moduli.

After mechanical testing, the samples were immersed in a mixture of formalin and ethylenediaminetetraacetic acid (EDTA) solution to fix and decalcify the samples, respectively. Subsequently, the samples were embedded in paraffin, followed by extraction of the histological sections of 3 µm thickness.

Polarized light microscopy (PLM) was utilized to measure the birefringence of collagen in the samples, which was used to quantify their collagen orientation. Unstained histological sections were placed on standard microscope slides and imaged with the Abrio PLM system (CRi, Inc., Woburn, MA, USA) fitted on a conventional light microscope (Nikon Diaphot TMD, Nikon, Inc., Shinagawa, Tokyo, Japan) [34]. Collagen orientation profiles were calculated in MATLAB (R2020b, MathWorks, Inc., Natick, MA, USA) by averaging a vertical profile from the superficial zone to the end of the deep zone. The angular threshold for separating between the superficial and middle zones and middle and deep zones were set at 20° and 70° [31], respectively. Average orientation in each of the zones was calculated, as well as their relative (%) thickness with respect to AC full thickness.

**Fig. 2:**
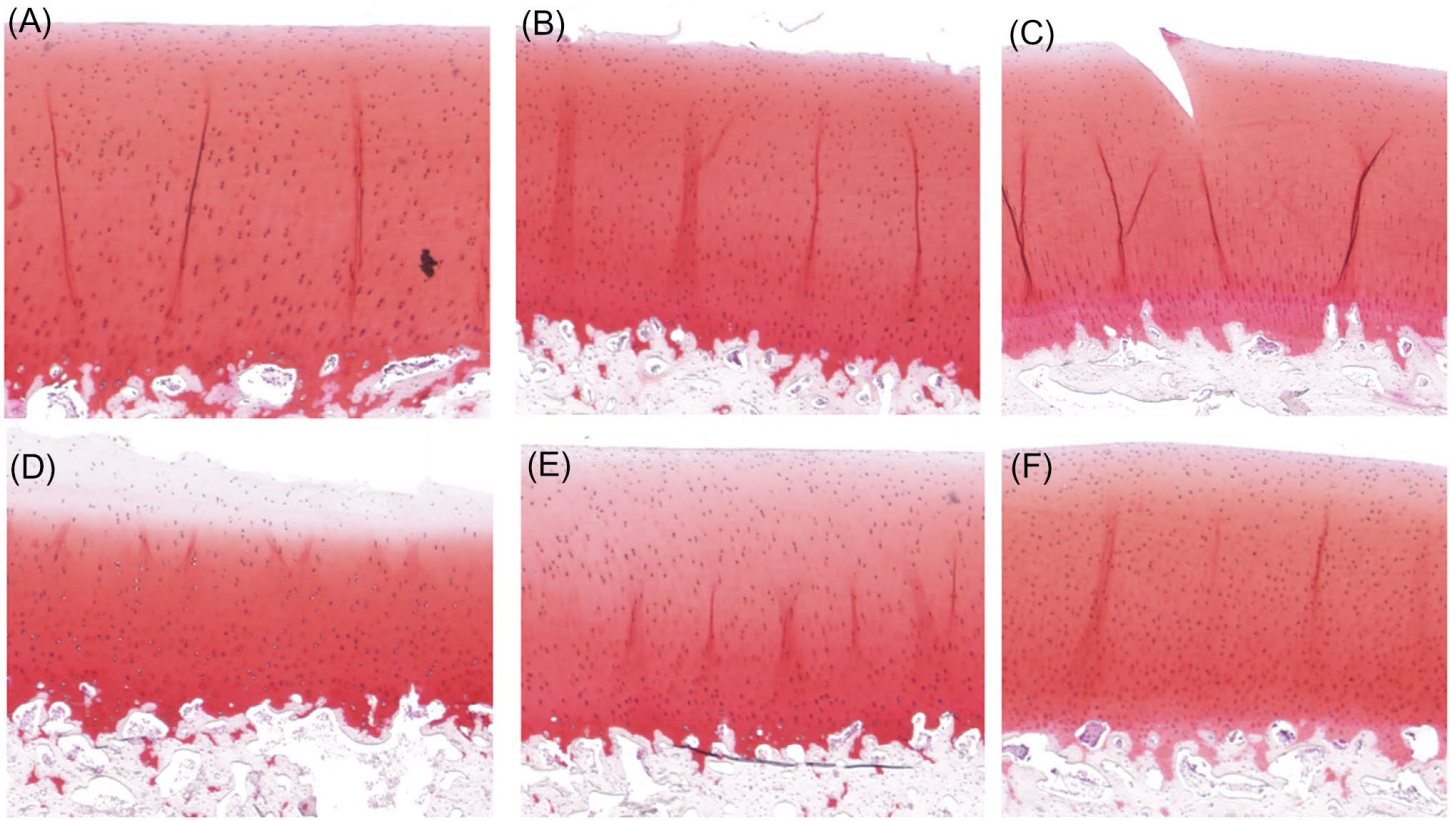
Safranin-O stained histological sections. **A** Healthy controls (CNTRL), **B** surface abrasion (SA), **C** impact loading (IL), **D** collagenase 24 h (COL24h), **E** collagenase 90 min (COL90m), and **F** trypsin 30 min (TRP30m)

Digital densitometry (DD) imaging was used to estimate the depth-wise PG distribution of the samples. Safranin-O, which stoichiometrically binds to PGs, was used to stain the previously prepared histological sections. The histological sections were then imaged with a PathScanEnabler-IV (Meyer Instruments, Inc., USA) [13], along with calibration filters having an optical density ranging from 0 to 3. The images were processed to obtain the average PG content in the different cartilage zones. The zones were determined from the relative (%) thickness calculations based on PLM measurements. Digital (RGB) images of representative samples from the distinct groups are presented in Fig. 2.

In total, thirteen reference properties were measured for each sample: three biomechanical properties (instantaneous, dynamic, and equilibrium moduli), seven structural properties (cartilage thickness, the collagen average orientation in the superficial, middle, and deep zone, the relative thickness of each cartilage zone), and three compositional properties (average PG content in the zones).

### Data Analysis

**Fig. 3:**
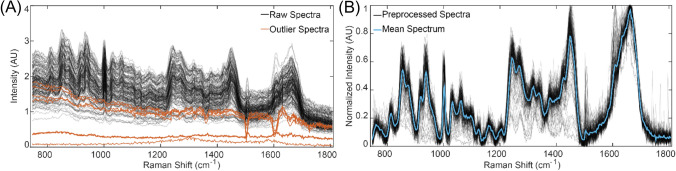
**A** Acquired raw spectra with outliers (fingerprint region). **B** preprocessed spectra with their mean

The acquired raw spectra (Fig. 3A) were visually inspected to remove outliers. Subsequently, the remaining spectra were pre-processed (Fig. 3B) by excluding data outside of the fingerprint region (750–1800 cm^−1^), baseline corrected—using asymmetrical least squares, and then min–max normalized using MATLAB. These pre-processing parameters were found to be optimal based on initial testing with other algorithms and parameters, such as derivative pre-processing and standard normal variate (SNV).

**Fig. 4:**
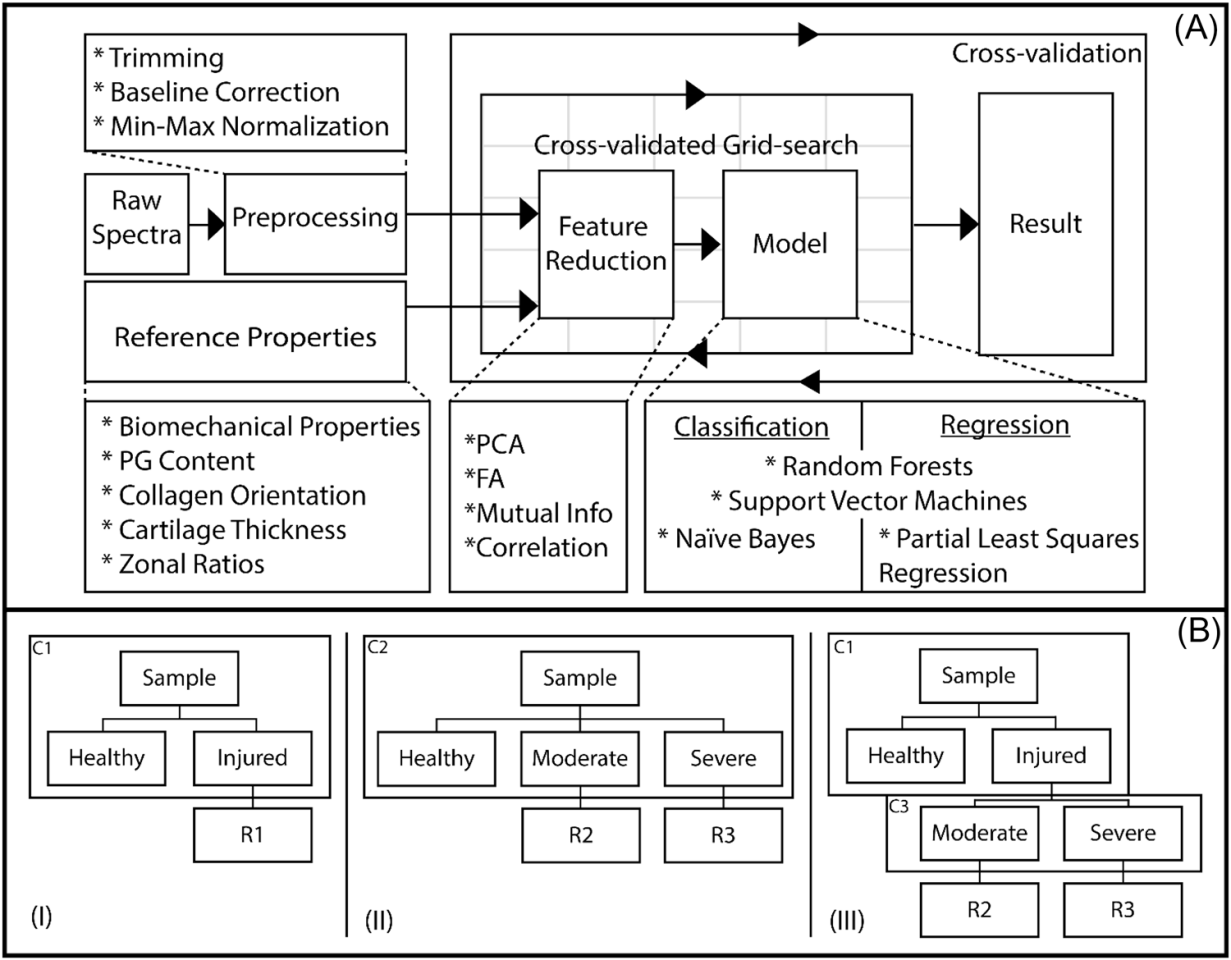
**A** Data analysis workflow, and **B** hybrid (classification-regression) machine learning pipelines for assessing cartilage integrity (*PG* proteoglycan, *PCA* principal component analysis, *FA* factor analysis)

Reference measurement values (target variables) for all samples were also checked for outliers using a median absolute deviation (MAD) criterion of more than three (>3) for the elimination of outliers. The pre-processed spectra with or without target variables (depending on the algorithm) were then passed to a feature reduction step (i.e., principal component analysis (PCA), factor analysis (FA), direct correlation (DC), and mutual information (MI) [35]), to reduce the dimensionality of the spectra. In each algorithm, features with the highest score or correlation were selected, varying from 1 to 20 selections. The selected features along with target variables were then passed onto the ML algorithms (Fig. 4A).

We adopted a two-stage approach for model development (Fig. 4B). In the first stage, a classifier was built to differentiate between healthy and degraded samples. In the second stage, a regression model was developed to estimate the tissue reference properties for the degraded samples.

While searching for the best approach for estimating AC properties, we investigated the option of classifying the artificially degraded samples into moderately and severely damaged classes, referring to previous literature that found that models tend to perform better when the two damage classes are separated, due to their considerable reference properties separation [8, 36]. We also developed simpler regression models based on the entire dataset representing the whole damage range, as a benchmark (C1, Fig. 4B-I).

In order to classify healthy, moderate, and severe damage classes, two approaches were implemented. The first approach involved the development of a simple multiclass classifier (C2, Fig. 4B-II), while the other approach involved a hierarchical two-step binary classifier (C3, Fig. 4B-III). For each reference property, three regression models: R1, R2, and R3 were developed (Fig. 4B). R1 was developed using the entire dataset (*n* = 165), while R2 and R3 were developed using only the moderately (*n* = 108) and severely (*n* = 57) degraded samples, separately.

Random forests (RF) and support vector machines (SVM) algorithms were considered for both regression and classification tasks, while Naïve Bayes (NB) was considered only for classification and partial least square regression (PLSR) only for regression tasks. These algorithms were chosen due to their superior performance with default parameters, relative to other classifiers and regressors of Scikit-learn [35]. A grid-search approach was used to tune and optimize each algorithm’s hyperparameters [37] (Table S1).

A nested cross-validation (CV) approach was adopted during ML analysis. In each iteration (6 in total), two groups (*n*_samples_ = 12) with controls and different degradations (IL, SA, COL90m, COL24h, and TRP30m) were left out for testing, while the remaining ten groups (*n*_samples_ = 60) were used for training and internal validation. A 5-fold CV was implemented in the training-validation step. F1-score was used for assessing the performance of the classification models, while normalized root-mean-square error (RMSE) and Spearman correlation (*ρ*) were the metrics of choice for assessing the performance of the regressors. RMSE was normalized by division over the target range. The median values of the test metrics were chosen to represent each model as the number of samples was limited. As each sample was measured three times, each sample had three predictions. For the final result, the median of these predictions was chosen in the case of regression and the mode in the case of classification. The best classification model was selected based on the highest *F*1-score; while in regression, the best model was selected by maximizing the *ρ*/RMSE ratio. The geometric mean of the test and CV metrics were used in this comparison.

MATLAB was used in statistical testing. The normality of reference distribution was determined via the Anderson–Darling test. Statistical significance of differences between groups was investigated using the Mann–Whitney *U*-test or unpaired *t*-test depending on the distribution with *p* < 0.05 as the limit for statistical significance.

## Results

Based on the spectral inspection and outlier statistics, four samples were completely removed from the analysis due to erroneous spectra (*n* = 2) or outlier reference properties (*n* = 2).

### Reference Properties

**Fig. 5:**
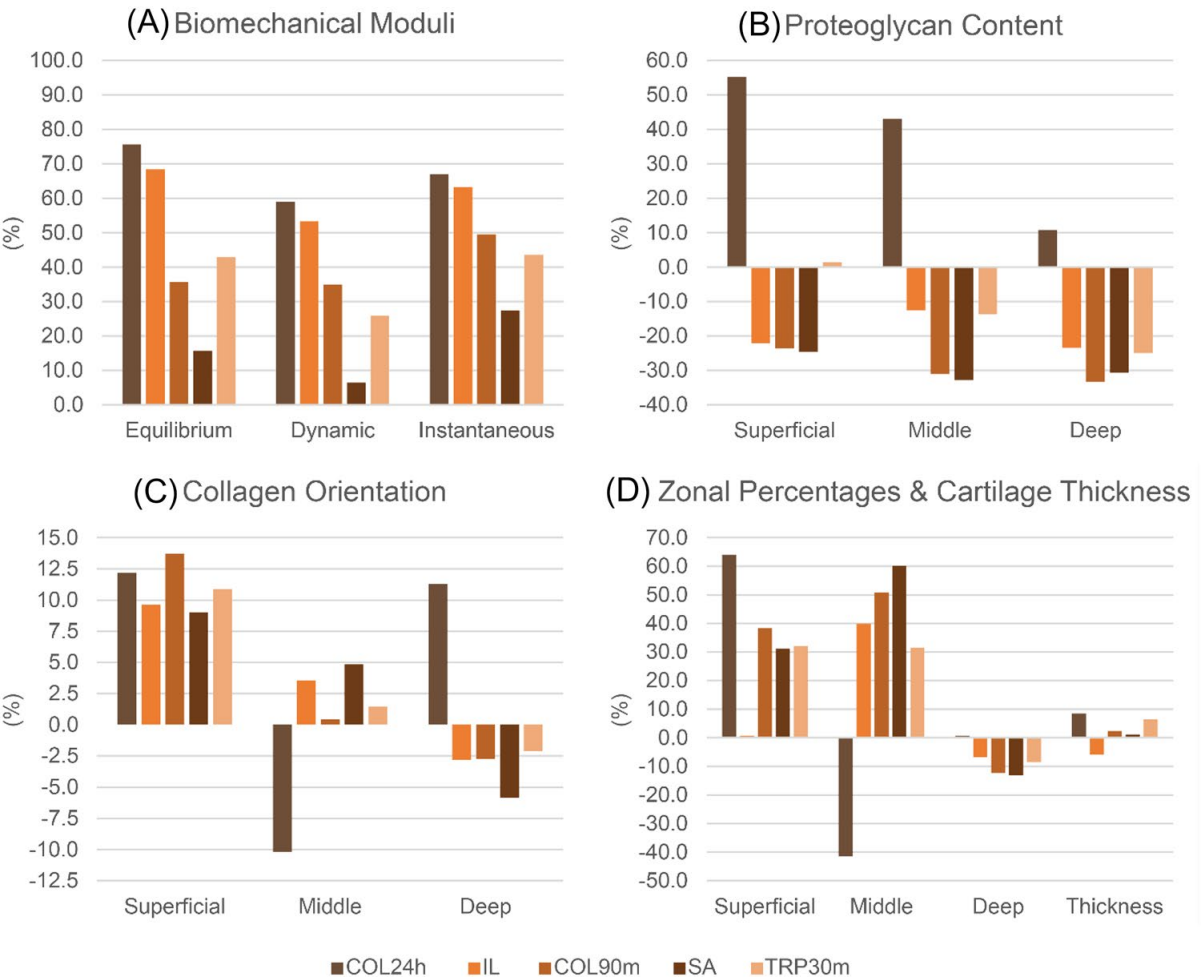
Percentage decrease in reference properties for each damage group relative to control group (*CNTRL* control, *IL* impact loading, *SA* surface abrasion, *COL24h* collagen 24 h, *COL90m* collagen 90 min, *TRP30m* trypsin 30 min)

Samples from the severe damage groups (COL24h and IL) exhibited the lowest values (*p* < 0.0001) for their biomechanical properties (Table 1) when compared to the other groups and to the control group (Fig. 5). In contrast, samples from the COL90m and TRP30m groups showed a moderate decrease in these properties, with samples from the SA group exhibiting the highest biomechanical properties (*p* < 0.0001). Regarding the PG content, CNTRL, COL24h, IL, and TRP30m groups exhibited lower values relative to other groups (*p* < 0.0001), with COL24h having the lowest values (*p* < 0.0001). It is worth noting that CNTRL samples exhibited PG content levels similar to (*p* = 0.097) degraded samples (Fig. 5), which can be attributed to location-dependent variations in cartilage composition as each group was taken from the same anatomic location. Collagen average orientation did not vary substantially between the damage groups (*p* > 0.2), except for the middle (*p* < 0.0001) and deep (*p* < 0.0001) zones of samples from the COL24h group. Lastly, the relative (%) thicknesses of each zone varied across all groups, particularly in the COL24h group.

**Table 1:** Statistics (mean ± standard deviation) of the reference properties for the five damage groups

Targets/groups	CNTRL	COL24h	IL	COL90m	SA	TRP30m
Equilibrium modulus (MPa)	1.13 ± 0.43	0.28 ± 0.09	0.36 ± 0.14	0.73 ± 0.32	0.96 ± 0.37	0.65 ± 0.30
Dynamic modulus (MPa)	6.51 ± 3.27	2.66 ± 2.48	3.04 ± 1.33	4.23 ± 0.87	6.08 ± 2.42	4.82 ± 1.32
Instantaneous modulus (MPa)	6.74 ± 3.37	2.23 ± 2.86	2.48 ± 1.02	3.40 ± 0.85	4.89 ± 1.94	3.80 ± 1.08
Thickness (mm)	1.908 ± 0.205	1.747 ± 0.369	2.021 ± 0.379	1.863 ± 0.315	1.887 ± 0.252	1.784 ± 0.176
Superficial zone (°)	12.01 ± 3.75	10.55 ± 6.56	10.86 ± 1.43	10.37 ± 2.72	10.93 ± 2.06	10.71 ± 2.53
Middle zone (°)	52.08 ± 3.08	57.38 ± 6.38	50.22 ± 2.64	51.84 ± 3.27	49.56 ± 1.61	51.33 ± 2.76
Deep zone (°)	77.14 ± 5.31	68.43 ± 25.91	79.32 ± 2.26	79.25 ± 3.11	81.65 ± 1.86	78.76 ± 3.63
Superficial zone (OD)	0.31 ± 0.18	0.14 ± 0.13	0.38 ± 0.19	0.38 ± 0.14	0.38 ± 0.21	0.30 ± 0.11
Middle zone (OD)	0.68 ± 0.34	0.39 ± 0.29	0.76 ± 0.25	0.89 ± 0.22	0.90 ± 0.24	0.77 ± 0.18
Deep zone (OD)	0.88 ± 0.25	0.79 ± 0.31	1.09 ± 0.15	1.18 ± 0.10	1.16 ± 0.18	1.10 ± 0.13
Superficial zone thickness (%)	7.80 ± 4.23	2.81 ± 3.58	7.74 ± 3.35	4.81 ± 2.10	5.37 ± 2.63	5.30 ± 3.10
Middle zone thickness (%)	13.26 ± 7.52	18.74 ± 27.46	7.98 ± 2.94	6.52 ± 2.05	5.29 ± 1.21	9.08 ± 5.31
Deep zone thickness (%)	78.95 ± 9.20	78.45 ± 30.60	84.28 ± 4.86	88.67 ± 2.68	89.34 ± 3.22	85.62 ± 7.72

*CNTRL* control, *COL24h* collagenase 24 h, *IL* impact loading, *COL90m* collagenase 90 min, *SA* surface abrasion, *TRP30m* trypsin 30 min

### Classification Tasks

**Fig. 6:**
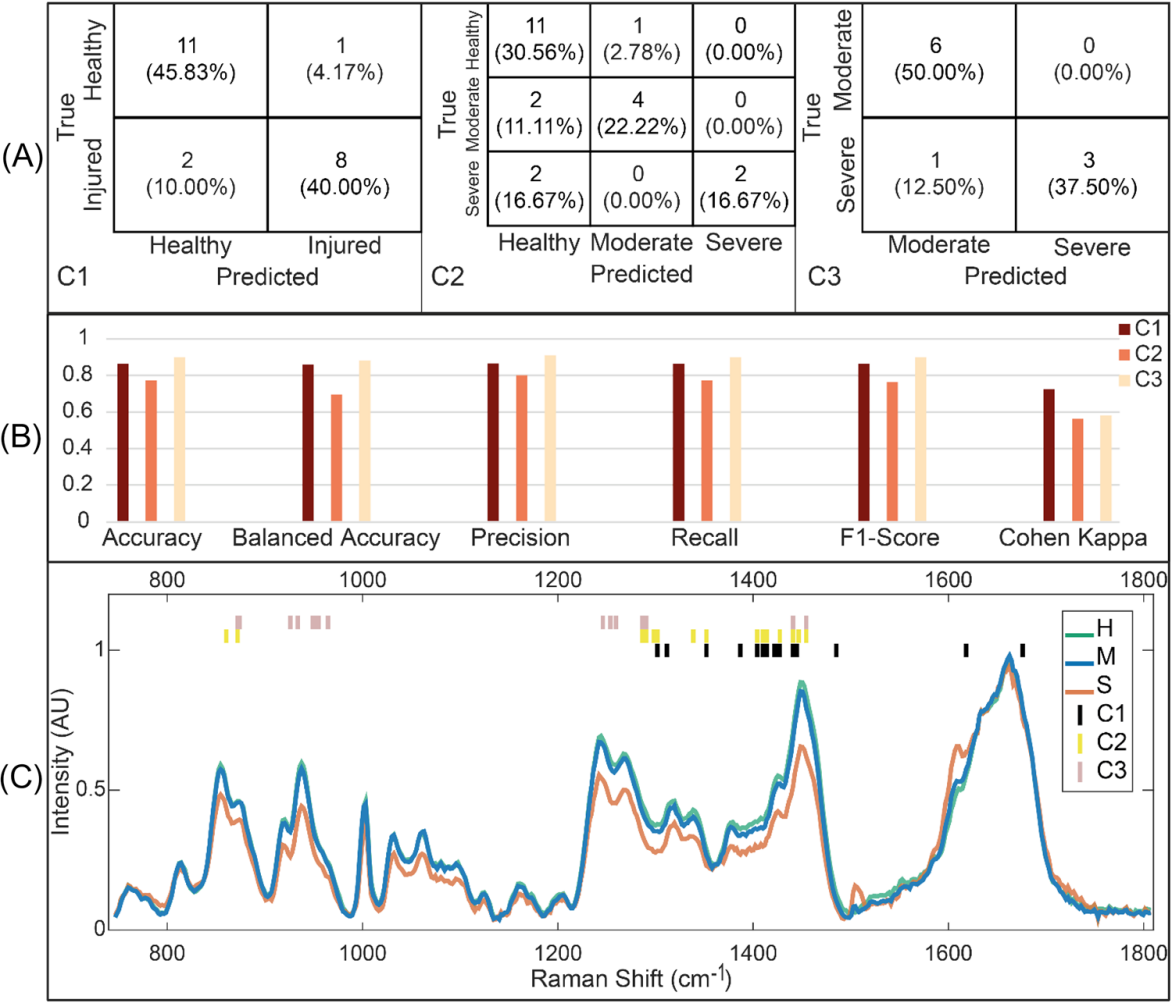
**A** Median confusion matrices of the best classification models (C1–C3, presented in Fig. 4B) for test set. **B** Test metrics of the classification models (C1–C3). **C** The mean spectra of healthy (H), moderately degraded (M), and severely degraded (S) samples in arbitrary units (AU), along with selected features of classifiers C1, C2, and C3

Classification models C1 and C3 performed better than C2 (Fig. 6A, B). Out of all possible combinations of feature selection (PCA, FA, DC, and MI) and classification (RF, SVM, and NB) algorithms, the best synergy was between MI and RF. All models utilized 15 features to arrive at the best separation. Features selected covered collagen and PG-related peaks, with the delta CH_2_/CH_3_ deformation band being consistently chosen across the three classification models. Other notable peaks include the proline band in classifiers C2 and C3 and 2 features across the width of the Amide I band in C1 (Fig. 6C).

### Regression Tasks

**Fig. 7:**
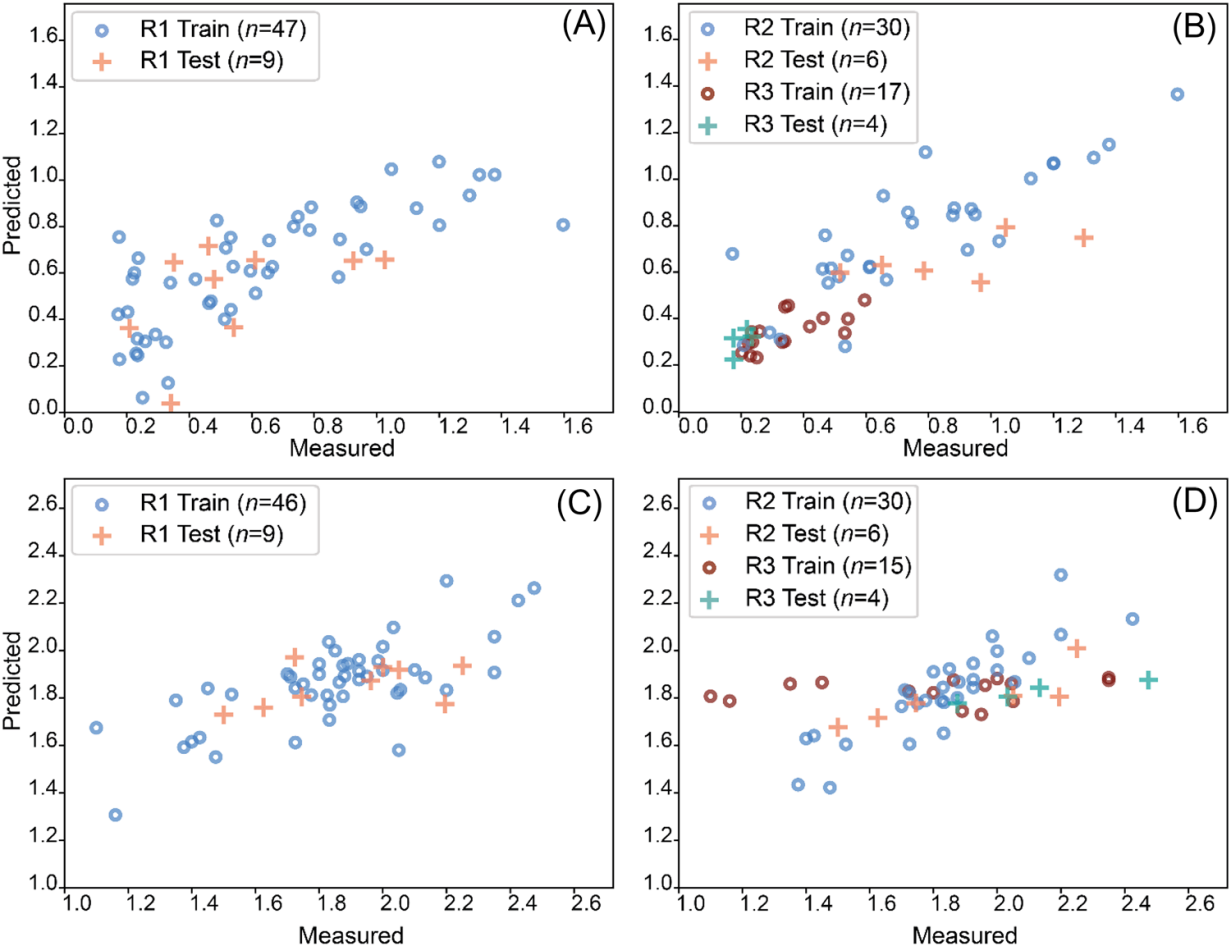
Scatter plots of the median performance for equilibrium modulus (MPa) (**A**, **B**) and cartilage thickness (mm) (**C**, **D**) with the best regression models [R1 (**A**, **C**) and the combination of R2 and R3 (**B**, **D**)]

Regression models for estimating the biomechanical and compositional properties demonstrated better correlation and lower errors compared to those specific to structural properties. Models for the prediction of PG content were the most consistent, exhibiting the best correlation (0.47 < *ρ* < 0.87) with relatively low error (14% < RMSE < 27%), followed by models for estimation of biomechanical properties (0.77 < *ρ* < 0.96, 12% < RMSE < 29%) and lastly collagen orientation (−0.24 < *ρ* < 0.79, 3% < RMSE < 9%) and zonal (%) thickness (0.33 < *ρ* < 0.47, 8% < RMSE < 29%). Specifically, models for estimating zonal properties (PG, CO, zonal (%) thickness) presented the lowest error in the deep zone followed by the middle zone and the superficial zone. In structural properties models, R1 (Fig. 7A, C) performed better than the combination of R2 and R3 (Fig. 7B, D; Table 2), while the opposite was true for compositional and functional properties.

**Table 2:** Spearman correlations and normalized root-mean-square error (RMSE) for test and cross-validation (CV) across R1 and the combination of R2 and R3

Property group	Targets	Regression 1		Regression 2 and 3	
		Spearman Test (CV)	RMSE % Test (CV)	Spearman Test (CV)	RMSE % Test (CV)
Functional Properties	Equilibrium modulus	0.62 (0.78)	15 (15)	0.85 (0.86)	18 (11)
	Dynamic modulus	0.70 (0.51)	24 (16)	0.96 (0.68)	29 (17)
	Instantaneous modulus	0.60 (0.42)	18 (12)	0.77 (0.71)	12 (15)
Compositional Properties	Proteoglycan Content				
	Superficial zone	0.41 (0.56)	18 (18)	0.47 (0.69)	21 (20)
	Middle zone	0.45 (0.86)	20 (19)	0.87 (0.90)	27 (16)
	Deep zone	0.72 (0.73)	11 (08)	0.82 (0.95)	14 (09)
Structural Properties	Collagen Orientation				
	Superficial zone	0.79 (0.44)	09 (16)	− 0.22 (0.55)	49 (23)
	Middle zone	0.55 (0.66)	03 (05)	0.47 (0.59)	26 (24)
	Deep zone	− 0.24 (0.12)	04 (04)	0.23 (0.57)	29 (18)
	Relative Thickness				
	Superficial zone	0.43 (0.68)	29 (17)	0.36 (0.80)	37 (13)
	Middle zone	0.33 (0.22)	08 (04)	0.19 (0.64)	16 (26)
	Deep zone	0.47 (0.96)	09 (02)	− 0.22 (0.85)	39 (10)
	Absolute thickness	0.47 (0.62)	09 (08)	0.90 (0.66)	27 (22)

## Discussion

In this study, we investigated the hypothesis that RS combined with ML provides an approach for discriminating between healthy and artificially degraded AC and inferring the tissue’s biomechanical, structural, and compositional properties. This proof-of-concept study established the basis for diagnostic assessment of cartilage integrity using RS and ML. Clinical translation of this technique in arthroscopic surgery can aid in the development of minimally invasive diagnostic tools for assessing cartilage health. Additionally, these tools potentially enable clinicians to make informed decisions about the most appropriate treatment for their patients. As a result, patient treatment outcomes can be improved. Moreover, the ability to accurately estimate biomechanical properties, PG content, collagen orientation, and zonal thickness can provide critical information for monitoring the efficacy of therapeutic interventions in the treatment of cartilage pathologies. Although few studies have demonstrated the capacity of other spectroscopic methods (e.g., NIR spectroscopy (NIRS)) [9, 10] for estimating AC properties, no other study, to the best of our knowledge, has attempted this with Raman spectra. Unlike other spectroscopic techniques, the surrounding fluid environment does not affect the Raman signal, making it an attractive technique for in vivo applications. Here, we were able to accurately classify healthy, moderately, and severely degraded AC samples, as well as estimate the tissue properties in vitro. The trained and validated ML models are important for the successful in vivo application of the technique as well as for advancing our understanding of the underlying mechanisms of cartilage degradation.

Classification models classifying two integrity states (i.e., binary models, such as healthy and degenerated samples) showed better accuracy than the three-state classification approach (multi-class, such as healthy, moderate, and severe groups), despite the three models showing reasonable errors. This can be attributed to the noticeable difference in spectral profiles between the combined healthy and moderate samples compared to severely damaged samples (Fig. 6C). This is evident from the metrics of degraded AC classification C3. On the other hand, most of the misclassifications observed in the multi-class model were moderately damaged samples misclassified as healthy and vice versa. It is worth noting that the high variability in the metrics, particularly in the test metrics, is likely due to the low number of samples.

The classification models compared the samples’ spectra based on specific wavenumbers, which were chosen using feature importance algorithms mentioned earlier. Out of the whole fingerprint region, wavenumbers identifying peaks of collagen and PGs along with the delta CH_2_/CH_3_ deformation band were consistently chosen in all three models. This deformation band was demonstrated by Beiroa et al. as an indirect index for assessing the progression of radiological OA [38]. For classifier C3 (moderate vs. severe), selected features comprised mostly of collagen-related peaks with the addition of phosphate-apatite peak [39], which is indicative of tissue mineralization—a process discriminating between moderate and severely damaged cartilage [18].

In our study, regression tasks showed varying degrees of success when predicting compositional and structural properties. Composition-related predictions (PG content and especially equilibrium modulus) were predicted with reasonable errors. On the other hand, large errors were observed when predicting structural-related properties in cartilage; although, RS has been shown to be sensitive to structural changes in biological samples [40, 41]. This may be related to sample fluorescence that could have masked the peak positional changes. Also, in retrospect, we believe the methodology to use the whole fingerprint region did not highlight the importance of peak positioning. Additionally, when comparing model performances in the three AC zones, properties in the deep zone were predicted with better accuracy than those from the superficial and middle zones, arguably resulting from the lower coefficient of variation (standard/mean) in the deep zone properties relative to the other two zones.

Previously, Shaikh et al. [13] have demonstrated the capacity of RS for discriminating between different types of artificial cartilage degradation with similar accuracy; in their paper, they presented a detailed comparison between pre and post degradation spectra for each damage group. However, our approach utilized a more rigorous nested CV approach as opposed to a single CV, which ensures that the choice of the training and test groups did not affect the results. Another study by Nippolainen et al. [8] attempted the same task albeit via NIRS and showed superior results. However, they used leave-one-out CV, which is a more optimistic approach.

Similar to our regression tasks, Sarin et al. [9] employed ensemble neural networks to relate the NIR spectra of cartilage to its collagen orientation, PG content, and thickness. For the superficial zone, the study reported inferior and similar results for collagen orientation and PG content, respectively, compared to the present study. For cartilage thickness, a lower prediction error was achieved; albeit the correlation was slightly lower than that of Sarin et al. [9] In addition, Sarin et al. [10] have evaluated the potential of NIRS for predicting cartilage biomechanical properties with better accuracy compared to the present study. However, Sarin et al. [10] used a total of five joints and 44 areas of interest, 41 out of which were used as training. Overall, RS showed a similar prediction performance to NIRS and essentially is a more suitable technique for in vivo applications.

Kroupa et al. [42] utilized a needle arthroscopic Raman probe for acquiring spectra, which were decomposed into composition-specific scores for GAGs, collagen, and water. In addition, polarized Raman spectra were collected to quantify collagen anisotropy in abraded cartilage samples. Their approach yielded models with *R*^2^ values of 0.95, 0.74, 0.94, and 0.90 for the prediction of GAG content, compressive modulus, indentation modulus, and cartilage thickness, respectively. Their derived Raman collagen alignment factor was significantly different for intact, mildly, and severely abraded cartilage samples. Overall, their work further supports our results on the ability of RS to estimate AC structural, compositional, and functional properties.

The main limitation of this study is the small number of samples relative to ML methods, like SVM and RF. Nevertheless, the sample size was sufficient [43] for establishing the potential of RS as a point-of-care for characterizing AC integrity in this proof-of-concept study. In addition, a Raman microscopy system, consisting of a 785 nm excitation laser, was used for spectral acquisition, resulting in spectra with high fluorescence and a relatively low signal-to-noise ratio (SNR). Increased exposure or number of accumulations would have risked overheating the sample. Nevertheless, we believe the low SNR did not influence study outcomes as the issue was mitigated in the pre-processing phase prior to ML analysis. It is also worth noting that recent developments in Raman instrumentation now allow for the suppression of the associated fluorescence signal, which may potentially enhance these results in future studies [44]. To mitigate the poor prediction performance of structural targets, two methods could be applied in future studies. The first of which involves the use of extracted peak information (amplitude, position, shift, standard deviation) rather than the whole fingerprint region. The second involves the utilization of polarized RS where structural changes are reflected more as stronger peak variations.

To conclude, RS is capable of discriminating between healthy and damaged cartilage, as well as estimating the tissue's functional, structural, and compositional properties with a reasonable error. The outcomes of this study demonstrate the clinical potential of RS for assessing AC integrity. Predicting AC properties would be beneficial for detecting the margin between healthy and diseased cartilage at the early stages of degeneration, where there may not be visually apparent signs of degradation. The proposed modality can potentially improve the arthroscopic examination of cartilage integrity.

### Supplementary Information

Below is the link to the electronic supplementary material.

Supplementary file1 (PDF 376 kb)
