# Supplementary material for: Raman Spectroscopy and Machine Learning Enables Estimation of Articular Cartilage Structural, Compositional, and Functional Properties
Source: Ann Biomed Eng. 2023 Jun 16;51(10):2301–12. doi: 10.1007/s10439-023-03271-5 (PMC10518284; doi:10.1007/s10439-023-03271-5)
Supplement: Supplementary file 1 — Supplementary file1 (PDF 376 kb) [file 10439_2023_3271_MOESM1_ESM.pdf]

Supplementary Material

For the manuscript:

Raman spectroscopy and machine learning enables estimation of articular cartilage structural, compositional, and functional properties

Eslam Shehata, Ervin Nippolainen, Rubina Shaikh, Ari-Petteri Ronkainen, Juha Töyräs, Jaakko K Sarin, Isaac O. Afara.

Table S1: Machine learning algorithms used with their hyperparameters search space.

Random Forest	Bootstrap	True, False
	Maximum Depth	80, 90, 100, 110
	Maximum Features	2, 3
	Minimum Samples Leaf	3, 4, 5
	Minimum Samples Split	8, 10, 12
	Number of Estimators	100
Support Vector Machines	Kernel	Linear, Poly, Sigmoid, Radial basis function
	C	0.001, 0.01, 0.1, 1, 10, 100, 1000
	Degree	1, 2, 3
	Gamma	Scale, Auto
Partial Least Squares Regression	Number of Components	1 - Number of Features
